# Atrial fibrillation, electroconvulsive therapy, stroke risk, and anticoagulation

**DOI:** 10.1186/s43044-023-00409-7

**Published:** 2023-11-27

**Authors:** Meera Kapadia, Pooja S. Jagadish, Marcus Hutchinson, Hong Lee

**Affiliations:** 1 Department of Internal Medicine at McGovern Medical School, University of Texas Health Science Center at Houston, 6431 Fannin, MSB 1.150, Houston, TX 77030 USA; 2 Division of Cardiology, Department of Medicine, University of Arizona-Tucson, 1501 N. Campbell Ave., PO Box 245046, Tucson, AZ 85724-5035 USA; 3 Department of Medicine, University of Arizona-Tucson, 1501 N. Campbell Ave., Tucson, AZ 85724-5035 USA

**Keywords:** Atrial fibrillation, Arrhythmia, Depression, Interdisciplinary, Psychiatry

## Abstract

**Background:**

Electroconvulsive therapy (ECT) is a therapy used to treat refractory mental health conditions, ranging from depression to catatonia, and it has gained renewed prominence in practice and the literature of late. Given that ECT involves the application of direct current to the body, there exists a risk of a change in cardiac rhythm during therapy. When atrial fibrillation is induced, ECT carries a potential risk of stroke. These risks have not been previously analyzed or summarized in the literature to allow physicians to make educated decisions about periprocedural risk and anticoagulation needs.

**Methods:**

To better describe this risk, the authors reviewed PubMed for articles that described the post-ECT cardioversion of AF to sinus rhythm, new development of AF post-ECT, and new stroke after either rhythm change.

**Results:**

Included were 14 studies describing 19 unique patients. Most patients had no rhythm change during at least one of many ECT sessions. Five patients converted from AF to sinus rhythm during at least one session, while AF followed ECT in seventeen patients during at least one ECT session. Four patients experienced both ECT-related cardioversion from AF to sinus rhythm as well as conversion from sinus rhythm to AF. Although no patients with a rhythm change experienced a stroke, one unanticoagulated patient who remained in AF developed a stroke post-ECT.

**Conclusions:**

Electroconvulsive therapy is demonstrated to be associated with rhythm changes—from atrial fibrillation to sinus rhythm as well as from sinus rhythm to atrial fibrillation. Thus, stroke risk during and after ECT remains a possibility. The anticoagulation of patients with AF who undergo ECT should be based on individual stroke risk factors, using validated stroke risk models, rather than prescribed routinely.

## Background

Electroconvulsive therapy (ECT) is a therapeutic option for treatment-resistant depression, bipolar disorder, and catatonia in patients [1, 2]. In ECT, a seizure is induced via the placement of two electrodes on the scalp in one of three major configurations: bilateral (bitemporal), right unilateral, and bifrontal [2]. Following the determination of each patient’s seizure threshold, and based on electrode position, a charge between 1.5 and 6 times the seizure threshold is delivered in pulses of up to 2.0 ms [2]. The conventional maximum charge is 576 mC in the United States and 1200 mC in the United Kingdom [3, 4]. The maximum energy delivered is approximately 100 J and has decreased with time [4]. In contrast, for the cardioversion of AF to sinus rhythm the initial energy requirement is 120–200 J for biphasic waveforms and 200 J for monophasic waveforms [5]. Pads are placed with a vector across the heart in one of three positions: anterior–posterior, anterior-left infrascapular, or anterior-lateral [5]. Per the guidelines, either patients are anticoagulated for 3 weeks before elective cardioversion or a pre-procedural transesophageal echocardiogram takes place to evaluate the left atrial appendage for thrombus [5, 6]. Although electrode positioning in ECT reduces the likelihood of charge delivery to non-cranial structures, the application of direct current and physiological changes associated with seizure induction may pose the risk of developing a new cardiac arrhythmia or, conversely, of cardioverting atrial fibrillation (AF) to sinus rhythm.

Through evolutions in procedural protocols and an improved safety profile, ECT was reclassified from Class III (high-risk) to Class II (moderate-risk, requiring special controls) in 2018 [2]. The use of ECT has been limited due to the potential adverse effects, including nausea, headache, apnea, hyperkalemia, prolonged seizure duration, and—more seriously—cardiac, pulmonary, and cerebrovascular events [1, 2]. Cardiac-specific events include asystole, hypertension, electrocardiographic abnormalities, transient wall motion abnormalities on echocardiogram, arrhythmias, and even myocardial infarction [1, 2]. By understanding the cardiac risks of ECT and learning how to mitigate them, the true role of ECT in patients who have refractory depression and other comorbid psychiatric conditions may be expanded. This study seeks to assess the risk of cardioversion of AF to sinus rhythm, of new development of AF from sinus rhythm, and of stroke risk in either of these groups. It further hopes to answer questions about the need for periprocedural anticoagulation and cardiology consultation.

## Methods

A search of the PubMed database was conducted independently and manually by two investigators to locate relevant studies through December 2022. Articles—primarily case reports and case series—were included if they described post-ECT cardioversion of atrial fibrillation to sinus rhythm, new stroke after ECT-induced AF cardioversion, and new development of AF after ECT. Studies were excluded if they lacked an English translation or did not specify a rhythm change involving atrial fibrillation. The primary outcomes were ECT-induced cardioversion of AF to sinus rhythm and incidence of post-ECT-related stroke in this population. The secondary outcome of interest was post-ECT AF induction and subsequent stroke rate. Results from each reviewer were compared, assessed for duplicate entries, and ultimately manually combined into Table 1 included in this manuscript.

**Table 1:** Summary of patient data

References	Age/sex	AF → SR	SR → AF	# ECT sessions
1. Craddock and Gilbert [10]	52 M		1	6
2. Harsch [7]	75 F	1	1	13
3. Hower and Yang [12]	70 M		1	38
4. Kuwahara et al. [17]	25 M		1	Not Specified
	46 M		1	
	74 M		1	
5.Loeffler and Capobianco [11]	45 M		1	> 25
6. Narasimhan [13]	70 F		1	1
7. O’Flanagan and Taylor [19]	54 M		1	10
8.O’Melia [14]	57 F		1	2
9. Olsen et al. [20]	21 M		1	Not Specified
10. Ottaway [9]	78 M	1		6
11. Petrides and Fink [8]	89 F	1	1	6
	76 F	1	1	7
	68 F	1		5
	64 M		1	6
12. Sattar et al. [18]	18 M		1	11
13. Urzal et al. [15]	64 M		1	> 2
14. Venditti et al. [16]	84 M		1	Not specified
	Total	5	17	

Table summarizing patient data from each of the 14 included manuscripts: age and sex of each patient, conversion from atrial fibrillation to sinus rhythm or sinus rhythm to atrial fibrillation with electroconvulsive therapy, and total number of electroconvulsive therapy sessions

*AF* atrial fibrillation, *SR* sinus rhythm, *ECT* electroconvulsive therapy

## Results

Included were 14 studies describing 19 unique patients. Five patients experienced cardioversion from AF to sinus rhythm at least once [7–9]. Four of these patients converted during the ECT session [8, 9], while one converted 60 s after the procedure [7]. One patient converted from AF to sinus rhythm on two separate occasions [8]. None of these patients developed a stroke. In total, these five patients underwent 37 ECT sessions, averaging 7.4 sessions each [7–9]. During most ECT sessions, these patients remained in AF. Demographically, four patients were older than 75 years, and all five were older than 65 years [7–9]. Four were female [7–9]. Three were taking tricyclic antidepressants at baseline, and two had unclear psychotropic medication history [7–9].

Seventeen patients developed new AF post-ECT [7, 8, 10–19], of whom no patients experienced a stroke. Many remained in AF transiently (up to 24 h), were cardioverted, or were medicated back to sinus rhythm using digoxin, beta-blockers, nicardipine, or amiodarone [7, 8, 10, 13, 15, 20]. Demographically, four were over 75 years old [7, 8, 16], seven were over age 65 [7, 8, 12, 13, 16, 17], and five were under age 50 [11, 17, 18, 20]. Five patients were female [7, 8, 13, 14], and sex was not specified for one patient [18].

Three patients experienced both conversion from AF to sinus rhythm as well as new development of AF on another ECT session [7, 8]. They were counted once in each category. The table lists each study and the outcome for the included patient(s): AF to sinus rhythm, sinus rhythm to AF, and number of ECT sessions (if available).

## Discussion

### The pathophysiology of conversion

ECT appears to be associated both with cardioversion from AF to sinus rhythm as well as the new development of AF in patients with baseline sinus rhythm [7–20]. However, the vectors used in ECT (across the brain) differ significantly from those used in synchronized cardioversion for AF (across the heart). In fact, current studies of the electrical field generated by ECT remain poorly defined but do not appear to include the body beyond the brain [21]. This suggests that there may be different pathophysiology for rhythm changes during or after ECT than solely the direct application of electrical current to the body.

One key explanation is that seizure induction affects circulating catecholamines and leads to an increased sympathetic tone [7, 9, 22]. The study by Jones and Knight [22] specifically assessed changes in catecholamines, blood pressure, and heart rate during ECT. They found, in the absence of beta-blockade, there were ischemic ECG changes, an increase in the rate pressure product (peak heart rate times systolic blood pressure) up to 35,000, a 15-fold increase in plasma epinephrine, and a three-fold increase in plasma norepinephrine. A rate pressure product of at least 30,000 is considered a sufficient workload to diagnose coronary disease in cardiac stress testing [23], so these patients effectively undergo a stress test during ECT. Furthermore, increased circulating catecholamines are thought to promote AF through a mechanism of increased magnitude of open L-type calcium channels as well as excessive intracellular calcium [24]. Thus, hyperadrenergic states via an effective stress test can logically lead to rhythm aberrancies.

Another plausible reason for rhythm conversion is the use of antiarrhythmic agents. Some of the patients who experienced cardioversion were also taking agents, including encainide, digoxin, and amiodarone, and one patient failed synchronized cardioversion events peri-procedurally; these factors improve the patient’s propensity to convert [7–9].

Notably, the use of many common psychiatric medications has known cardiovascular conduction effects. Although the concern is primarily for QT-prolongation and ventricular events [7, 8], combined with periprocedural anesthetic use, these medications can alter physiologic thresholds, facilitating rhythm changes [7]. Thus, the mechanism of conversion is multi-faceted.

### Conversion to sinus rhythm

ECT led to the cardioversion of AF to sinus rhythm in five cases. A majority of the patients who experienced cardioversion to sinus rhythm were elderly with multiple cardiovascular comorbidities, and no cardioversion events led to a stroke in the time these patients were under the care of the respective manuscript’s authors—a duration that was not consistently specified.

The five patients who converted from AF to sinus rhythm are as follows: Harsch [7] described a 75-year-old female with hypertension and AF diagnosed 10 months prior. She spontaneously converted to sinus rhythm 60 s after an ECT session and converted from sinus rhythm to AF on two, separate, later occasions. Ottaway [9] describes a 78-year-old M with coronary artery disease status post 4-vessel coronary artery bypass grafting who developed post-operative stroke, ventricular tachycardia, and atrial fibrillation. He was in AF and anticoagulated with warfarin when he presented for ECT. After his second session, he developed an unspecified, symptomatic tachyarrhythmia associated with pallor and hypotension, so he was started on amiodarone. This event prompted synchronized cardioversion for AF before the next session. It was unsuccessful, so he was started on amiodarone, and ECT proceeded without issue. He subsequently converted from AF to sinus rhythm during the 4th ECT session.

Last, Petrides and Fink [8] describe the conversion of AF to sinus rhythm in three patients: (1) An 89-year-old female with congestive heart failure and AF who was trialed on multiple antidepressant and antipsychotic medications without improvement in her depression. Following her first session of ECT, she converted from AF to sinus rhythm, and during a later session, she reverted to AF. (2) A 76-year-old female with coronary artery disease, congestive heart failure, aortic valve replacement, and hypertension was incidentally found to have new AF pre-ECT, so she was started on warfarin. She alternated between AF and sinus rhythm during her second session and converted to sinus rhythm during her third session. (3) A 68-year-old female with AF and subacute bacterial endocarditis converted to sinus rhythm after her first session of ECT. She subsequently underwent four unremarkable ECT sessions.

### Development of atrial fibrillation

There are 17 reported cases of AF following ECT. Three of these cases were in patients who previously converted from AF to sinus rhythm, as described earlier [7, 8]. At least another three cases relate to pre-treatment with the antipsychotics olanzapine, clozapine, and quetiapine, which affect cardiac conduction and repolarization. A 70-year-old male treated with olanzapine had previously tolerated ECT but developed AF after 38 uneventful treatments [12]. Another, a 64-year-old male patient treated with clozapine, developed AF following his second ECT [15]. This case of AF induction was additionally attributed to the use of a charge of 230 mC, 500% greater than his seizure threshold. Narasimhan [13] documented a 70-year-old female taking quetiapine who developed AF with rapid ventricular response and 4-mm diffuse T-wave inversions immediately following bifrontal 50% ECT. This patient converted back to sinus rhythm after administration of esmolol and magnesium sulfate; however, further investigation was required to understand the etiology of her abnormal cardiac findings. Chart review revealed a 200-pack-year history of tobacco use and critical stenosis of his left anterior descending artery. Narasimhan postulated that pre-existing cardiac abnormalities likely contributed to arrhythmia propagation following ECT.

Kuwahara et al. [17] described 3 patients with catatonic schizophrenia who each converted 10–14 min after ECT. A 25-year-old male was pretreated with atropine due to the potential for asystole when ECT is performed on the nucleus of the vagus nerve. This synergistic effect of atropine and ECT stimulation was thought to lead to the development of AF. A 46-year-old and 74-year-old male were both incidentally noted to go into AF 10 min post-ECT, and it was postulated by the authors to be related to “delayed and marked sympathetic excitation...as a consequence of ECT” in patients with catatonic schizophrenia, in particular [17].

Next, AF induction appears to primarily occur during the second and subsequent ECT sessions. Venditti et al. [16] describe an 84-year-old male with hypertension who developed AF after 4 years of bimonthly ECT sessions. Similarly, Loeffler and Capobianco [11] report of a 45-year-old otherwise healthy male patient experienced AF after 25 treatments [11]. Craddock and Gilbert [10] describe a 52-year-old male who experienced AF during his second ECT treatment with a return to normal sinus rhythm spontaneously after three hours. O’Melia [14] describes a 57-year-old male who developed AF during his second ECT session and spontaneously converted back to sinus rhythm after 36 h. Petrides and Fink [8] report on a 64-year-old female with known atrial flutter who developed atrial fibrillation on her sixth session, requiring treatment with digoxin. Last, O’Flanagan and Taylor [19] describe a 54-year-old M who developed documented AF after his 8th session and an irregular pulse following his 9th session, but a confirmatory ECG was not available.

Sattar [18] performed a study of the electrocardiogram (ECG) changes after ECT in 25 patients, noting frequent sinus tachycardia (72% of patients) and one episode of AF induction in an 18-year-old otherwise healthy patient. The patient spontaneously reverted to sinus rhythm after 8 days and without treatment. Olsen et al. [20], describes a 21-year-old male patient who had previously undergone multiple, uneventful ECT sessions but developed AF treated with synchronized cardioversion during one session [20]. Majority of the studied patients undergoing ECT sessions remain in sinus rhythm. Remaining in sinus rhythm (normal rate or tachycardic) is the most common outcome.

### Stroke risk and bleeding complications

It is important to consider that many of the included cases predate the widespread use of validated stroke and bleeding risk models and do not reliably disclose the risk factors that physicians currently consider when assessing stroke and bleeding risk, such as hypertension and diabetes. Even though patients may not have been appropriately anticoagulated by today’s standards, only one thromboembolic event was described by Suzuki et al. [25]. A 77-year-old female with hypertension and chronic AF who was non-adherent to anticoagulation suffered an embolic stroke one day after her 43rd ECT session, despite remaining in AF post-ECT. She underwent all 42 ECT sessions without cardioversion or other systemic consequences. This is the only known, published case of a patient with AF developing a stroke post-ECT [25], and it is not related to cardioversion of AF.

Intracranial hemorrhage is a potential—but exceedingly rare—complication of ECT [8, 13, 25]. No intracranial or systemic major bleeding events were described in the case studies included in this review.

### ECT and AF: should we anticoagulate?

Stroke risk with cardioversion can be mitigated by appropriate periprocedural anticoagulation, but anticoagulation in ECT still poses concerns for major bleeding events, such as intracranial hemorrhage, despite the relative rarity. Several studies have examined safe anticoagulation use in patients with AF who undergo ECT [8, 26–28]. Warfarin and direct oral anticoagulants (DOAC) such as apixaban, rivaroxaban, edoxaban, or dabigatran were analyzed, and they were determined to be safe without major bleeding events post-ECT [8, 26–28]. In fact, the patient described by Suzuki et al. [25] may well have avoided stroke had she been adherent to an anticoagulation regimen.

Based on this review, the best course of action is to follow the latest guidelines on atrial fibrillation to guide not only stroke- and bleeding risk assessment but also to assist with anticoagulant choice for each patient based on individual risk factors. Guidelines currently favor DOACs as first-line for stroke prevention in AF, excepting patients with mechanical heart valves, very recent implantation of bioprosthetic material, and valvular AF, highlighting lower rates of bleeding complications associated with DOACs than warfarin [6, 29, 30]. The management of patients who must be on warfarin should follow the latest relevant guidelines (e.g., valvular and atrial fibrillation) to guide target INR selection, and these patients should maintain guideline-direct follow-up intervals with an anticoagulation clinic. Overall, the is no clear contraindication to anticoagulating patients with AF who are at risk for embolic stroke based on validated stroke risk models [6]. In contrast, there is no evidence to support routine anticoagulation before ECT.

### Pre-ECT evaluation

Pre-ECT evaluation should follow a similar protocol to those in the most recent guidelines for perioperative cardiovascular evaluation prior to noncardiac surgery [31], bearing in mind that perioperative risks and those associated with ECT are somewhat different. The main similarities include the use of anesthesia and significant periprocedural hemodynamic shifts.

Evaluation should start with a careful history and physical exam performed by a member of the patient’s mental health team. Preemptive consultation of a primary care provider, hospitalist, or cardiologist for risk stratification (akin to preoperative evaluation) is not necessary, would delay care, and would add healthcare expenses to the patient and system. During this initial visit, a patient’s history should be evaluated for prior arrhythmias or arrhythmic symptoms, such as palpitations, chest discomfort, lightheadedness, or weakness. History of cardiovascular implantable electronic devices should be noted, ensuring that a cardiologist is involved in its management. For patients who use clinically validated wearable technology, such as the Apple Watch [32, 33], it is pertinent to ask if an irregular heartbeat has ever been detected. This must be followed by a review of the corresponding rhythm strip(s) to reduce the risk of misdiagnosis from artifact or non-AF irregular rhythms [29, 32]. Of note, current AF guidelines emphasize caution with the use of wearables because many devices are not clinically validated [6, 29]. The 2020 European Society of Cardiology guideline for the diagnosis and management of AF somewhat allows for the use of wearables, stating they can be used to definitively diagnose AF if a “single-lead ECG tracing of ≥ 30 s or 12-lead ECG” demonstrates AF—as reviewed by a “physician with expertise in ECG rhythm interpretation” 29. Non-ECG-based wearables (photoplethysmography) cannot be used to make the diagnosis and would require additional testing. Thus, data from wearable technologies should not be neglected as part of the history. The cardiovascular physical exam should include palpation of bilateral peripheral pulses for rate and rhythm, auscultation for murmurs and gallops, and assessment of blood pressure to improve overall risk stratification and prompt further evaluation if the examination is concerning for AF.

If the history and physical exam is negative for suspicion of arrhythmia, then no further testing would be necessary. This will avoid cascade testing that could, at best, delay necessary treatment for the patient and, at worst, lead to complications from testing. Following the model of preoperative risk stratification, routine 12-lead ECG or echocardiography would not be indicated [31].

If the history and physical exam raise concern for an arrhythmia, a 12-lead ECG should be obtained. If the 12-lead ECG reveals AF or atrial flutter, then initiation of anticoagulation should be based on the most recent atrial fibrillation guidelines [6]. It may be appropriate to consult medicine or cardiology for anticoagulation recommendations and risk stratification, depending on the scope of practice and comfort level of the healthcare team members performing the initial evaluation. If outpatient referral is placed for risk stratification and anticoagulation management prior to the initiation of appropriate anticoagulation, patients would be without appropriate therapy for the duration of the wait for a referral appointment. This may be an extended time, and the patient could have a thromboembolic event in the interim. Thus, using convenient guideline-directed [6] risk stratification tools, such as CHA_2_DS_2_-VASc [34], can facilitate appropriate initiation of anticoagulation at the initial evaluation visit where history and physical examination was performed. This process is more streamlined if the patient is hospitalized, since consultants can provide timely recommendations, but a new diagnosis of AF should not prompt hospitalization for an otherwise outpatient procedure.

If the 12-lead ECG does not reveal AF or atrial flutter, it is imperative to weight the risks and benefits of further testing and referrals. Downstream testing may include arrhythmic monitoring, such as a Holter monitor (24–48 h) or event monitoring (up to 30 days). This would delay ECT. Considering that this literature review demonstrated a single stroke in a non-anticoagulated patient who underwent ECT, the risk of thromboembolic events does exist among patients who are not on optimal therapy, but this risk is relatively low. Discussion with an interdisciplinary team, including medicine and cardiology may be helpful to further elucidate risk, especially if ECT is non-urgent. Wearable tools, such as the Apple Watch, may detect an irregular heart rhythm but often do not correlate with AF or atrial flutter [32, 35]. As stated previously, a diagnosis of AF *can* be made by clinically validated ECG-based devices, but uncertainty of the rhythm should be followed up with additional ECG-based recordings, such as a 12-lead ECG or Holter monitor [29]. Wearables should not be routinely used for diagnosis. Although routine use of these wearables has led to increased diagnosis of AF, the clinical significance remains questionable [32, 35]. Thus, until the guidelines comment further on use of these tools, they have limited role in evaluation beyond assessment of irregular heart rhythm in the patient’s history.

Among patients with AF or atrial flutter who are newly started on anticoagulation and have an urgent indication for ECT (within 30 days), there is insufficient data to either recommend or advise against TEE or cardiac computed tomography. However, the overall risk of thromboembolic events based on this review is low. The risk of further evaluation through structural imaging can increase patient cost, delay care, and lead to incidental findings. The pursuit of these should be personalized to each patient and their cardiac risk factors. Shared decision-making with the patient and interdisciplinary discussion that includes a cardiologist may be considered.

### Study limitations

There are several limitations to this literature review. Since the data is derived from a small number of case reports and case studies—six of which are at least thirty years old—it may be difficult to generalize this information to broader, more-modern populations when the ECT dose delivered has decreased. Additionally, home medications and patient comorbidities are not consistently documented. Some psychotropic medications (particularly tricyclic antidepressants) have effects on cardiac conduction, which can affect outcomes. Furthermore, antecedent data on asymptomatic, historical AF cannot always be known, and AF is generally prevalent in the study population. Last, ECT dose also was not consistently reported, and this may affect catecholaminergic surge and propensity for cardioversion. Without documentation of predisposing factors, it remains possible that AF and ECT timing is coincidental.

### Future areas of study

This study seeks to provide a starting point for additional research in the field, and several topics require further investigation. To specifically quantify risk, a large, randomized study that assesses patients with known AF for cardioversion to sinus rhythm post-ECT is warranted. Additional research on the catecholaminergic effects of ECT may also elucidate an underlying mechanism for rhythm changes. Last, with improvements in wearable cardiac technology since the publication of the majority of these cases, a screening process may evolve to include ambulatory cardiac event monitoring before ECT. In addition to anticoagulation safety in ECT, the role of pre-screening for AF would be an area for future study since there are no current recommendations on the topic.

## Conclusions

Among patients with underlying atrial fibrillation who undergo electroconvulsive therapy for the management of psychiatric disorders, there is a demonstrable risk of cardioversion to normal sinus rhythm with a non-negligible risk of embolic stroke, regardless of conversion to sinus rhythm. Furthermore, ECT is associated with AF development with theoretical stroke risk. There is insufficient evidence to support the routine anticoagulation of all patients before ECT; however, physicians should engage in shared decision-making about initiating anticoagulation in those patients who have baseline AF or develop AF post-ECT; this discussion should be based on known stroke risk factors to best mitigate the risk of post-procedural embolic stroke.

## Data Availability

The list of manuscripts included—as obtained through PubMed review—are referenced within the manuscript and summarized in Table 1.

## Abbreviations

AF: Atrial fibrillation
DOAC: Direct oral anticoagulants
ECT: Electroconvulsive therapy
ECG: Electrocardiogram

## References

[1] Andrade C, Arumugham SS, Thirthalli J (2016). Adverse effects of electroconvulsive therapy. Psychiatr Clin N Am.

[2] Espinoza RT, Kellner CH (2022). Electroconvulsive therapy. N Engl J Med.

[3] Krystal AD, Dean MD, Weiner RD (2000). ECT stimulus intensity: Are present ECT devices too limited?. Am J Psychiatry.

[4] Treatment Procedures (2008) In: The practice of electroconvulsive therapy: recommendations for treatment, training, and privileging: a task force report of the American Psychiatric Association, 2nd ed. American Psychiatric Association, p 368 10.1097/PRA.0000000000000914 41894194

[5] Neumar RW, Otto CW, Link MS (2010). Part 8: adult advanced cardiovascular life support: 2010 American Heart Association Guidelines for Cardiopulmonary Resuscitation and Emergency Cardiovascular Care. Circulation.

[6] January CT, Wann LS, Calkins H et al (2019) AHA/ACC/HRS focused update of the 2014 AHA/ACC/HRS guideline for the management of patients with atrial fibrillation: a report of the American College of Cardiology/American Heart Association Task Force on Clinical Practice Guidelines and the Heart R. Heart Rhythm. 10.1016/j.hrthm.2019.01.024 10.1016/j.hrthm.2019.01.024 30703530

[7] Harsch HH (1991). Atrial fibrillation, cardioversion, and electroconvulsive therapy. Convuls Ther.

[8] Petrides G, Fink M (1996). Atrial fibrillation, anticoagulation, and electroconvulsive therapy. Convuls Ther.

[9] Ottaway A (2002). Atrial fibrillation, failed cardioversion, and electroconvulsive therapy. Anaesth Intensive Care.

[10] Craddock WL, Gilbert HP (1948). Transient auricular fibrillation complicating electric shock therapy. Am J Psychiatry.

[11] Loeffler G, Capobianco M (2012). Resuming electroconvulsive therapy (ECT) after emergence of asymptomatic atrial fibrillation during a course of right unilateral ECT. J ECT.

[12] Hower MR, Yang C (2020) New onset paroxysmal atrial fibrillation after electroconvulsive therapy. Cureus. 10.7759/cureus.11717 10.7759/cureus.11717 PMC7772109 33391948

[13] Narasimhan S (2008). Electroconvulsive therapy and electrocardiograph changes. J Postgrad Med.

[14] O’Melia J (1970). A case of atrial fibrillation following the use of suxamethonium during ECT. Br J Psychiatry.

[15] Urzal F, Oliveira-Maia AJ, Barahona-Correa JB (2019). Acute-onset atrial fibrillation following an electrically induced generalized convulsion in a patient treated with clozapine and electroconvulsive therapy. J ECT.

[16] Venditti RC, Shulman MS, Lutch SB (1992). Atrial fibrillation after electroconvulsive therapy. Anaesthesia.

[17] Kuwahara T, Yoshino A, Horikawa N, Nomura S (2009). Delayed sympathetic hyperactivity following electroconvulsive therapy in patients with catatonic schizophrenia. Nihon Shinkei Seishin Yakurigaku Zasshi.

[18] Sattar SA, Rajendra P, Sreenivas N (1992). Electrocardiographic changes following electroconvulsive therapy. J Assoc Phys India.

[19] O’Flanagan PM, Taylor RB (1950). Auricular fibrillation after electric convulsion therapy. Br Med J.

[20] Olsen B, Trent JS, Inman BL. Electroconvulsive Therapy-Induced Paroxysmal Atrial Fibrillation in Healthy Young Male. *Cureus*. 2022;14(11):e31989. 10.7759/cureus.31989 10.7759/cureus.31989 PMC9797374 36589202

[21] De Deng Z, Lisanby SH, Peterchev AV. Electric field strength and focality in electroconvulsive therapy and magnetic seizure therapy: A finite element simulation study. J Neural Eng. 2011;8(1):016007. 10.1088/1741-2560/8/1/016007 10.1088/1741-2560/8/1/016007 PMC3903509 21248385

[22] Jones RM, Knight PR (1981). Cardiovascular and hormonal responses to electroconvulsive therapy. Modification of an exaggerated response in an hypertensive patient by beta-receptor blockade. Anaesthesia.

[23] Berman JL, Wynne J, Cohn PF (1978). A multivariate approach for interpreting treadmill exercise tests in coronary artery disease. Circulation.

[24] Workman AJ (2010). Cardiac adrenergic control and atrial fibrillation. Naunyn Schmiedebergs Arch Pharmacol.

[25] Suzuki H, Takano T, Tominaga M, Suzuki K, Kagaya Y (2010). Acute embolic stroke in a patient with atrial fibrillation after electroconvulsive therapy. J Cardiol Cases.

[26] Centanni NR, Craig WY, Whitesell DL, Zemrak WR, Nichols SD (2021). Safety of ECT in patients receiving an oral anticoagulant. Ment Health Clin.

[27] Shuman M, Hieber R, Moss L, Patel D (2015). Rivaroxaban for thromboprophylaxis in a patient receiving electroconvulsive therapy. J ECT.

[28] Schmidt ST, Lapid MI, Sundsted KK, Cunningham JL, Ryan DA, Burton MC (2014). Safety of electroconvulsive therapy in patients receiving dabigatran therapy. Psychosomatics.

[29] Hindricks G, Potpara T, Dagres N (2021). 2020 ESC Guidelines for the diagnosis and management of atrial fibrillation developed in collaboration with the European Association for Cardio-Thoracic Surgery (EACTS): The Task Force for the diagnosis and management of atrial fibrillation of the European Society of Cardiology (ESC) Developed with the special contribution of the European Heart Rhythm Association (EHRA) of the ESC. Eur Heart J.

[30] Writing Committee Members, Otto CM, Nishimura RA et al (2020) ACC/AHA guideline for the management of patients with valvular heart disease: a report of the American College of Cardiology/American Heart Association Joint Committee on Clinical Practice Guidelines. J Thorac Cardiovasc Surg 162(2):e183–e353. 10.1016/j.jtcvs.2021.04.002 10.1016/j.jtcvs.2021.04.002 33972115

[31] Eagle KA, Berger PB, Calkins H et al (2002) ACC/AHA guideline update for perioperative cardiovascular evaluation for noncardiac surgery—executive summary. A report of the American College of Cardiology/American Heart Association Task Force on Practice Guidelines (Committee to Update the 1996 Guidelines on Perioperative Cardiovascular Evaluation for Noncardiac Surgery). Anesth Analg 94(5):1052–1064. 10.1097/00000539-200205000-00002 10.1097/00000539-200205000-00002 11973163

[32] Seshadri DR, Bittel B, Browsky D (2020). Accuracy of apple watch for detection of atrial fibrillation. Circulation.

[33] Perez MV, Mahaffey KW, Hedlin H (2019). Large-scale assessment of a smartwatch to identify atrial fibrillation. N Engl J Med.

[34] Lip GYH, Nieuwlaat R, Pisters R, Lane DA, Crijns HJGM (2010). Refining clinical risk stratification for predicting stroke and thromboembolism in atrial fibrillation using a novel risk factor-based approach: the euro heart survey on atrial fibrillation. Chest.

[35] Perino AC, Gummidipundi SE Lee J et al (2021) Arrhythmias other than atrial fibrillation in those with an irregular pulse detected with a smartwatch: findings from the apple heart study. Circ Arrhythm Electrophysiol 14(10):e010063. 10.1161/CIRCEP.121.010063 10.1161/CIRCEP.121.010063 34565178

